# Maladaptive Eating in Children and Adolescents With Obesity: Scrutinizing Differences in Inhibition

**DOI:** 10.3389/fpsyt.2020.00309

**Published:** 2020-04-16

**Authors:** Tiffany Naets, Leentje Vervoort, Ann Tanghe, Ann De Guchtenaere, Caroline Braet

**Affiliations:** ^1^Clinical Child and Adolescent Psychology (CCAP) Lab, Department of Developmental, Personality and Social Psychology, Ghent University, Ghent, Belgium; ^2^Obesity Department, Zeepreventorium vzw, De Haan, Belgium

**Keywords:** childhood obesity, external eating, inhibition, impulsivity, executive functions, self-control

## Abstract

**Introduction:**

In order to grasp the complex etiology of childhood obesity, we aim to clarify the relationship between external eating and weight. Based on theory and empirical evidence, we claim that inhibition is an important moderator in this association. In our first research question we expected that high external eating would be related to a higher weight status, especially for those with high inhibition problems. Secondly, we explored the moderating role of inhibition in the association between external eating and weight change after a multidisciplinary obesity treatment.

**Method:**

We investigated n=572 participants (51% boys, aged 7–19) with moderate to extreme obesity recruited in a Belgian inpatient treatment center. At intake, parents reported on inhibition (BRIEF), while the children and adolescents reported on their eating behavior (DEBQ). Weight and length were objectively measured pre and post treatment (ADJUSTED BMI). Two hierarchical linear regression models were built to scrutinize the influence of inhibition on the association between external eating and both baseline weight and weight change.

**Results:**

First, predicting baseline weight, we found no significant moderating effect of inhibition problems. Second, predicting weight loss, inhibition turned out to be a substantial moderator, specifically in adolescents. Some unexpected gender differences occurred in favor of adolescent boys, in a way that those with high external eating and low inhibition problems lost most weight.

**Conclusion:**

Inhibition problems act as a moderator explaining weight loss, but this only holds for adolescents. This suggests that external eating and inhibition play a complex role in weight loss in certain age and gender categories, and stresses the importance of identifying subgroups for tailoring interventions. For those with high inhibition problems, interventions aimed at increasing inhibition skills might be needed to optimize treatment outcomes.

## Introduction

Obesity portends risks for lifelong medical and psychosocial problems in millions of children and adolescents worldwide (1). The accumulation of fat, the main characteristic of overweight and obesity, is due to an energy imbalance in which intake exceeds expenditure (2). The etiology driving this imbalance is complex, although it is recognized that psychological processes initiating maladaptive eating behavior could be seen as a crucial contributor (3).

External eating is an important type of maladaptive eating behavior, defined as food-driven eating in which physiological homeostasis is ignored (4, 5). Schachter and colleagues (6, 7) already posit that an external orientation is a personality trait and refer to external eating as food intake in response to external cues—such as smell, taste, and appearance—overruling internal bodily signals such as hunger and satiety. This mechanism can easily be triggered by an obesogenic environment, where the abundant availability of palatable food provides a constant flow of tempting food cues (4, 8, 9). It has been frequently shown that weight is increased in children, adolescents, and adults who show high external eating patterns, in comparison to individuals without this maladaptive eating behavior (10–15).

Evidence is rising on the role of self-control deficits in explaining why some, but not all children and adolescents with high external eating develop overweight (15–17). External eating can be better understood by an in depth study on the role of Executive Functions (EFs), the most well-known processes to achieve top-down self-control in response to the environment (18, 19). The three main EFs are inhibition, cognitive flexibility, and updating working memory. They refer to the capacity of inhibiting impulses, shifting flexibly between tasks or mind-sets, and processing and retrieving up-to-date information (20). EFs are known to develop through the lifespan. They gradually improve throughout childhood—parallel with cognitive development and through adequate challenges posted by parents, schools, and relevant others—reaching maturation in late adolescence (21). It is known that the first signs of simple inhibitory capacities emerge at a young age (approximately at the age of 3), and that complexity increases (22). In adolescence, approximately at the age of 14, inhibition gradually becomes interwoven in networks of other executive functions (23, 24).

There is ample evidence on the role of EFs, and mainly inhibition, as determinants of (un)controlled eating behavior and weight status (25). In separate studies, inhibition problems are found to be related to (1) more external eating, (2) a higher weight status, and (3) more difficulties to lose weight. First, maladaptive eating behavior in adults is found to be at least partially explained by lower inhibition, which manifests itself as impulsive behavior (16, 26). Impulsivity seems to explain why some people cannot resist external food cues (26–29), not only in adults with obesity, but also in adolescents of varying weight (30). Consistently, children with overweight who are highly impulsive, consume more palatable foods in comparison to those with lower impulsivity (31). However, evidence linking inhibition with eating behaviors in children and adolescents with obesity remains scarce (30, 31). Second, inhibition is also found to be a determinant of weight status in both adults (32, 33), children (34) and adolescents (35, 36), with higher problems associated with higher weight. Third, inhibition also appears to influence weight change trajectories throughout treatment. High impulsivity hinders weight loss, in both adults (37), children (38), and adolescents (39). When inhibitory control is trained, a decrease of inhibition problems can predict a better treatment outcome (40–42).

Despite these separate arguments on the role of inhibition affecting eating behavior at one hand, and weight status (weight gain and weight change) on the other, the reciprocal associations between these processes are still unclear (14). Scrutinizing the role of inhibition in this association, especially in children and adolescents, is an important and much needed new direction in research. Furthermore, given the evidence that inhibition can be modified and trained through computerized tasks, increased knowledge offers possibilities for improving obesity treatment (19, 43–45).

In sum, we can state that there is a clear gap in the knowledge on how inhibition affects excessive responding to external cues and how that is related to weight status and weight change in children and adolescents with obesity. The aim of the present study is to expand insights into the link between this maladaptive eating behavior and weight (both baseline weight status and weight change after treatment), hypothesizing that inhibition is a moderator in these relationships. First, we hypothesize that more external eating will predict a higher weight status, and that this relationship will be stronger for those with more inhibitory control deficits. Second, we will explore whether more external eating before treatment will predict weight change after treatment, and if this association will be moderated by inhibition. Although we do not have specific hypotheses, we will explore whether gender plays a role in the moderation. When studying youngsters, we have to take developmental factors into account as well. Since inhibition gradually develops in childhood and matures in adolescence, we expect that these relationships might be different for children and adolescents.

## Materials and Methods

### Participants

This clinical sample consisted of 572 youngsters with obesity between 7 and 19 years old (mean age = 14, SD = 2.39), in which 51% were boys. The mean adjusted BMI at baseline of the sample was 187 (SD = 30.9). The child group within this sample consisted of 220 children aged 7 to 13 (M = 11, SD = 1.5), in which 56% were boys. Their mean adjusted BMI at baseline was 186 (SD = 29.6). The adolescent group consisted of 352 youngsters aged 14–19 (M = 16, SD = 1.3), in which 48% were boys. Their mean adjusted BMI at baseline was 189 (SD = 31.7). The child and adolescent group did not differ in weight status (*F*(1,568) = 1.22, p =.173) or gender (*X^2^* (1,572) = 3.38, *p* =.066). All participants were recruited from a Belgian inpatient center, providing Multidisciplinary Obesity Treatment (MOT) for youth with moderate to severe obesity. Dutch and French speaking youngsters between 7 and 19 years old were included, with normal intelligence, and identified as primarily obese (46). Treatment drop-out was 5%. Study attrition, determined as a non-completion of (one of) the questionnaires was 29%.

The evidence-based MOT program focused on changing lifestyle behavior by providing a healthy diet, daily physical activity, and a psychological cognitive behavior therapy (CBT) program (46, 47). The inpatient setting installs a stable environment with minimized exposure to food and maximum opportunities for physical activity, supporting children and adolescents with severe obesity who cannot achieve substantial weight loss in their own obesogenic home environment (46). Age appropriate interventions are offered in separate child and adolescent therapy groups. Guidance is provided by a multidisciplinary team of dieticians, pediatricians, physiotherapists, psychologists, and social workers. They provide parent education sessions as well. The program consists of an introduction, intermediate and consolidation phase spread over twelve months (46). Youngsters receive education at the facility’s school, and partially return home during weekends and holidays. A more detailed description of the program can be found in Braet et al. (46).

Prior to the start of data-collection, both the participants and their parents provided active informed consent. The study was approved by the faculty ethical committee (2015/88), and principles concerning privacy and ethical research were respected in accordance to national laws and the Declaration of Helsinki and its later amendments of 1964. The Belgian implementation of the General Data Protection Regulation (GDPR) of May 25th 2018 is the current standard.

### Measures

#### BRIEF Behavior Rating Inventory of Executive Function

Parents reported on inhibition problems of their children, through a 75-item questionnaire on a three-point Likert scale (ranging from 1 to 3: “never” to “often” a problem). Higher scores represent more EF problems. The BRIEF (48) consists of eight subscale scores (inhibition, flexibility, emotional control, initiation, working memory, planning/organizing, and monitoring), but for this study only the scores on the inhibition subscale were used (for example “*My child has trouble putting the brakes on his/her actions*” or “*My child does not think before doing [is impulsive]*”). Higher scores indicate more inhibition problems. To compare inhibition scores of the participants to inhibition scores in an age and gender appropriate Belgian norm group, and to increase interpretability, standardized T-scores were calculated based on the specific norm groups of Belgian peers within the same gender group (48). T-scores are considered as “clinical” when scoring 60 or higher (48). This instrument received good psychometric evaluations (48, 49). Internal consistency of the inhibition scale in the present study was good (Cronbach’s *α* =.87).

#### DEBQ Dutch Eating Behavior Questionnaire

Children reported on their own external eating behavior through a 33-item questionnaire on a five-point Likert scale (ranging from 1 to 5: occurring “never” to “very often”). Higher scores represent more maladaptive eating. The DEBQ (9) consists of three subscales (external, emotional, and restrained eating), but for this study the scores on the external eating subscale were used (for example “*If food smells and looks good, do you take a bigger portion than usually?*” or “*If you pass the baker do you want to buy something tasty?*”). Higher scores indicate more external eating. Standardized T-scores were used in order to compare external eating behavior scores to those in the norm population (10). External eating can be considered “clinical” when scoring 60 or higher (9). This instrument received good psychometric evaluations (13, 50). Internal consistency of the external eating scale in the present study was good (Cronbach’s α = .87).

#### Adjusted BMI

To index weight status and weight change in a developmentally appropriate way (51), the Adjusted BMI (ABMI) was used to take into account age and gender differences amongst BMI scores (52). The standard BMI was calculated by dividing calibrated weight by squared height. This was then divided by the mean (Percentile 50) for that specific age range and gender in the norm group (53). By multiplying by 100, it can be interpreted as a percentage of overweight: 120–140% is considered as overweight, 140–160% as moderate obesity, and 160%+ as severe to extreme (52). Next, differences between baseline weight (pre) and weight after MOT (post) were calculated, representing a percentage of weight change in comparison to the initial weight status calculated *via* the following formula: ΔABMI = (ABMI pre − ABMI post)/pre ABMI × 100 (54).

### Procedure

Over the years (from 2013 to 2017), several waves of participants were recruited. They completed an online questionnaire tool at the beginning of treatment during intake (baseline, pre). Weight and length data were collected by a pediatrician or dietician, before (on the same day as the questionnaire tool) as well as after MOT (post). Participants and their parents could receive assistance during data collection, especially to help the young children complete the questionnaires.

### Data Analysis

We tested the interaction between external eating and inhibition in predicting 1) baseline weight status and 2) weight loss after Multidisciplinary Obesity Treatment (MOT) using multiple linear regression. Prior to model testing, we looked at the percentages of participants showing high, moderate, and low problems (based on T-scores categories) of external eating, inhibition, and a combination of both.

For theoretical reasons and in order to facilitate interpretation, we dichotomized age and analyzed the models for children (7–13 years) and adolescents (14–19 years) separately. The cut-off between children and adolescents is based on the age where there is a theoretical and empirical observable difference between childhood and adolescent manifestation of inhibition, also reflected in the age cut-off point in the BRIEF norm groups (23, 48).

Model 1 contained a hierarchical linear regression of three blocks predicting baseline weight status (ABMI): (1) the main effects of external eating (DEBQ external eating subscale T-scores), inhibition problems (BRIEF inhibition subscale T-scores) and gender (boy/girl) were included in the first block (2) in block two we added two-way interactions between external eating and inhibition, external eating and gender, and inhibition problems and gender, and (3) the final block consisted of the three-way interaction between external eating, inhibition problems and gender. Model 2 contained the same blocks, predicting weight change after MOT (ΔABMI).

Three assumptions were tested *a priori* for each model: multicollinearity (by the Pearson correlations between the predictors, VIF, and Tolerance statistics), the independency of errors (by the Durbin-Watson statistic), and normal distribution (by plotting standardized residuals and Kolmogorow-Smirnov statistics). Data were analyzed using IBM SPSS Statistics 24.

## Results

### Descriptives

Sample descriptives can be found in **Table 1**. The subsample descriptives showed differences in subgroup percentages of those scoring high and low on external eating and/or high or low inhibition problems.

**Table 1:** External eating, inhibition, and our weight parameters in the (sub)sample(s).

	Total sample	Children		Adolescents	
		Boys	Girls	Boys	Girls
N	572	123	97	169	183
Mean Age (SD)	14 (2.4)	11 (1.3)	11 (1.5)	15 (1.2)	15 (1.2)
Baseline weight	187.82	186.32	184.67	190.64	187.93
Mean ABMI pre (SD)	(30.88)	(32.41)	(25.59)	(34.45)	(28.9)
Weight change	−25.82	−28.07	−26.44	−27.48	−22.59
Mean ABMI change (SD)	(10.28)	(9.81)	(10.88)	(10.78)	(9.0)
External Eating (EE)					
Low EE (< 40)	18.5%	25.2%	17.7%	24.2%	12.4%
Moderate EE (40–60)	53.8%	53.9%	55.2%	58%	57.6%
High EE (≥ 60)	22.9%	20.9%	27.1%	17.8%	29.9%
Inhibition Problems (INH)					
Low INH (< 40)	23.6%	29.3%	17.5%	20.7%	25.7%
Moderate INH (40–60)	56.8%	55.3%	57.7%	58%	56.3%
High INH (≥ 60)	19.6%	15.4%	24.7%	21.3%	18%
EE & INH					
Low EE and low INH	6.6%	9.8%	4.1%	8.9%	3.8%
Low EE and high INH	3.1%	1.6%	3.1%	4.1%	3.3%
High EE and high INH	5.9%	2.4%	8.2%	5.3%	7.7%
High EE and low INH	3.8%	4.1%	4.1%	2.4%	9.2%

### Model 1: Predicting Baseline Weight Status

Assumption testing showed no substantial signs of violation. Correlations between the predictors never exceeded.90 (max *r* =.77 for external eating correlated to inhibition). Although tolerance statistics violated the cut-off rule of.20, the variation inflation factor statistic always stayed below 10 (max *VIF* = 2.773 in block 4 for external eating * inhibition). Errors were independent, with the Durbin-Watson parameter situated between 1 and 3 (*DW* = 2.03) in block 4. The Kolmogorow-Smirnov statistic was significant (*p* < .001), but normal distribution can be assumed by the central limitation theorem, and also the plots (regression of standardized residuals) showed no clear signs of violation of the Normality assumption (55).

Regression analysis (**Table 2**) revealed no significant predictors of weight status, neither in children nor in adolescents. The theoretically premised interaction between external eating and inhibition problems (*β*= −.02, *t* = −.795, *p* = .428 in children, *β*= −.01, *t* = −.464, *p* = .643 in adolescents), nor the other two-way interactions, nor the main effects, significantly predicted baseline weight status.

**Table 2:** *Model 1:* Hierarchical linear regression predicting ABMI in children and adolescents.

Block		B	SE B	β
Children				
1	(Constant)	190.8	11.12	
	EE	−.11	.15	−.05
	INH	.01	.18	.005
	Gender	−1.4	4.07	−.02
2	(Constant)	168.6	37.46	
	EE	.16	.72	.08
	INH	.52	.77	.20
	Gender	10.67	22.95	.18
	EE × INH	−.01	.01	−.21
	EE × Gender	.11	.31	.10
	INH × Gender	−.35	.37	−.31
3	(Constant)	195.41	50.44	
	EE	−.38	.99	−.17
	INH	−.04	1.05	−.02
	Gender	−46.37	75.39	−.79
	EE × INH	.01	.02	.15
	EE × Gender	1.23	1.44	1.12
	INH × Gender	.82	1.52	.74
	EE × INH × Gender	−.02	.03	−1.13
Adolescents				
1	(Constant)	182.32	9.8	
	EE	.16	.14	.06
	INH	.01	.15	.004
	Gender	−3.67	3.46	−.05
2	(Constant)	155.92	31.15	
	EE	.60	.62	.23
	INH	.29	.58	.11
	Gender	27.77	19.83	.44
	EE × INH	−.004	.01	−.11
	EE × Gender	−.43	.29	−.38
	INH × Gender	−.19	.31	−.15
3	(Constant)	171.12	45.22	
	EE	.28	.92	.11
	INH	−.003	.86	−.001
	Gender	.35	62.32	.006
	EE × INH	.003	.02	.07
	EE × Gender	.11	1.21	.10
	INH × Gender	.35	1.19	.29
	EE × INH × Gender	−.01	.02	−.50

(1) EE, External Eating; INH, Inhibition problems.

(2) in children, R² =.003 for block 1, ΔR² =.006* for block 2, ΔR² =.003* for block 3. In adolescents, R² =.006 for block 1, R² =.009* for block 2, ΔR² =.001* for block 3. (*) p < .10, *p < .05; ** p < .01, *** p < .001.

### Model 2: Predicting Weight Change After MOT

Assumption testing showed no substantial signs of violation. Correlations between the predictors never exceeded.90 (max *r* =.77 for external eating correlated to inhibition). Although tolerance statistics violated the cut-off rule of .20, the variation inflation factor statistic always stayed below 10 (max *VIF* = 2.82 in block 4 for EE x INH). Errors were independent, with the Durbin-Watson parameter situated between 1 and 3 (*DW* = 2.15) in block 4. The Kolmogorow-Smirnov statistic was violated (*p* < .001) and residual plots slightly showed positive skewness, but normal distribution can be assumed by the central limitation theorem (55).

**Figure 1:**
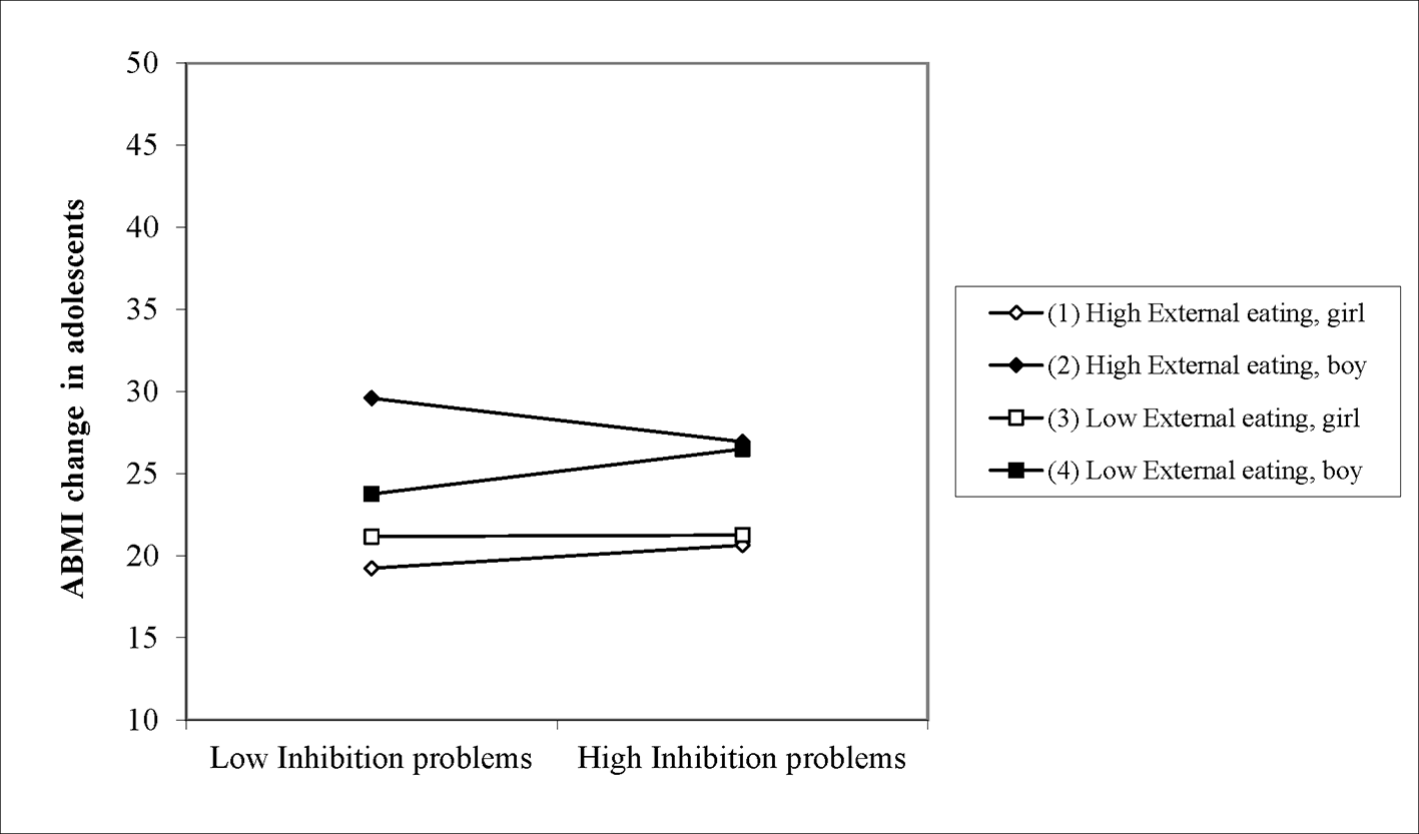
Three-way interaction predicting ABMI change in adolescents.

Regression analysis (**Table 3**) in children showed no significant predictors for weight change. Regression analysis on weight change in adolescents revealed main effects of inhibition problems (*β*= 0.504, *t* = 1.875, *p* = .06) and external eating (*β*= 0.64, *t* = 2.239, *p* < .05) and a significant interaction between external eating and gender (*β*= −.79, *t* = −2.08, *p* < .05). The three-way interaction between external eating, inhibition problems and gender did not reach conventional limits of significance (*β*= 0.12, *t* = 1.731, *p* = .08), but suggests that the two-way interactions (e.g., external eating × gender or external eating × inhibition problems, (*β*= −.01, *t* = −1.78, *p* = .07), should be interpreted with caution. As can be seen in **Figure 1**, adolescent boys with low inhibition problems and high external eating lost most weight.

**Table 3:** *Model 2:* Hierarchical linear regression predicting ΔABMI in children and adolescents.

Block		B	SE B	β
Children				
1	(Constant)	21.83	3.98	
	EE	.07	.05	.09
	INH	.06	.07	.07
	Gender	−2	1.46	−.10
2	(Constant)	3.77	13.39	
	EE	.41	.26	.53
	INH	.473	.27	.51
	Gender	−4.76	8.2	−.23
	EE × INH	−.01	.005	−.71
	EE × Gender	.08	.11	.21
	INH × Gender	−.03	.13	−.07
3	(Constant)	−2.67	17.91	
	EE	.54	.35	.70
	INH	.61	.37	.65
	Gender	9.2	26.99	.44
	EE × INH	−.01	.007	−.95
	EE × Gender	−.19	.51	−.51
	INH × Gender	−.31	.55	−.81
	EE × INH × Gender	.01	.01	.80
Adolescents				
1	(Constant)	21.68	3.09	
	EE	.06	.05	.08
	INH	.06	.05	.06
	Gender	−5.08	1.11	−.25
2	(Constant)	12.15	9.80	
	EE	.28	.19	.33
	INH	.16	.18	.18
	Gender		6.25	−.002
	EE × INH	−.04	.004	−.24
	EE × Gender	−.003	.091	−.42
	INH × Gender	−.15	.10	.14
3	(Constant)	−5.36	14.07	
	EE	.64*	.29*	.77*
	INH	.50(*)	.27(*)	.56(*)
	Gender	31.97	19.52	1.58
	EE × INH	−.01(*)	.01(*)	−.90(*)
	EE × Gender	−.79*	.38	−2.16*
	INH × Gender	−.57	.38	−1.48
	EE × INH × Gender	.01(*)	.01(*)	1.84(*)

(1) EE, External Eating; INH, Inhibition problems.

(2) in children, R² =.02 for block 1, ΔR² =.01* for block 2, ΔR² =.0.001* for block 3. In adolescents, R² =.07 for block 1, ΔR² =.009* for block 2, ΔR² =.008* for block 3. (*) p < .10, *p < .05; ** p < .01, *** p < .001.

## Discussion

Obesity in children and adolescents is a serious condition, with a complex etiology that until today is not fully understood (2, 3). In the present study, we aimed to increase the knowledge on psychological processes driving weight status and weight change during treatment. External eating, or eating in response to external cues instead of physiological signals, is an important maladaptive behavior contributing to a higher weight (6, 10, 11). Unfortunately, there are several gaps in the knowledge on how external eating is related to obesity. Research in children and adolescents on this topic is limited, and we still do not fully understand why some external eaters develop overweight while others do not. Furthermore, it is unknown if and how external eating contributes to weight loss during treatment. Recent research points to the role of Executive Functions (EFs), an umbrella term for top-down self-control processes (18, 20). EFs gradually develop through childhood and still mature until late adolescence (21). EFs are proven to be flawed in both children, adolescents, and adults with obesity (45, 56). Especially youngsters with low inhibition, often described as highly impulsive or having high inhibition problems, are assumed to be unable to control their external eating (16, 26). They have a higher weight (33, 34), and they also seem to have more difficulties to lose weight (25, 38). Taken together, there are several separate arguments that obesity is at least partially influenced by external eating, and that limited inhibition seems to exaggerate the problem. This fuels our hypothesis of the moderating role of inhibition for understanding unhealthy eating habits in weight problems. Unfortunately, these relationships are still unclear, especially in children and adolescents with obesity. That is why the present study investigates inhibition as a moderator in the relation between external eating, and both baseline weight status and weight change after evidence-based Multidisciplinary Obesity Treatment (MOT). Because of the ongoing maturation of inhibitory capacities (21), we expected differences when studying this interaction between external eating and inhibition in children and adolescents. Moreover, we also explored potential differences between boys and girls without *a priori* hypotheses. After all, on a descriptive level, our data showed clearly that there are age group and gender differences in levels of external eating, inhibition problems, and the degree of weight loss after MOT.

In the first hierarchical model, predicting baseline weight in children and adolescents separately, we formally tested if high external eating was associated with higher baseline weight, and whether this relationship was especially strong for those with high inhibition problems. Our hypothesis was not confirmed. We could not show that external eating, inhibition problems nor the interaction contribute to baseline weight in children and adolescents with obesity. This means that, based on weight observations only, we cannot know whether problems with external eating, inhibition, or both are at play in explaining baseline weight. It should be noted that the baseline weight status was extremely high in this sample. This could explain why we were unable to support the hypothesis that individual differences in external eating and/or inhibition determine individual differences in ABMI. After all, it is known that as weight increases, more determinants—such as socio-economic status (57, 58) and parental involvement (57, 59)—play a complicated role and are increasingly interfering with the well-known personal factors and biological determinants (60, 61).

However, interestingly, in the second series of analyses on psychological determinants associated with weight change, differences could be detected. In the second hierarchical model, predicting weight change after inpatient treatment in children and adolescents, we evaluated whether external eating was associated with weight change, and if this relationship was moderated by inhibition. Although inhibition problems are known to hinder weight loss (38), little is known on how external eating in itself influences weight change and how inhibition relates to that. The results of our analysis showed that most weight loss was observed in adolescent boys with low inhibition problems and high external eating. Although this finding seems surprising, we have to acknowledge the important role of the inpatient environment for these children and adolescents during treatment. The inpatient MOT is offered in a highly controlled and healthy environment for a long period (47). This context, with strong restrictions on food cues and food availability, could be considerably helpful for those with high external eating at baseline. For those less sensitive for external cues, the contextual effect of this specific inpatient environment might be less pronounced. Our observation that weight loss is highest in adolescents with strong inhibitory capacities (in combination with high external eating), dovetails with earlier studies showing that inhibition does play a role in which inhibition problems are known to hinder treatment (38) and stronger inhibition predicts more favorable treatment outcomes (40, 41). These findings plead for separate interventions aiming at increasing inhibition in all youngsters with high inhibition problems: in order to equip those highly reactive to the obesogenic environment (i.e., those with high external eating) with the necessary skills to resist the temptation, we need to offer them additional training to strengthen their inhibitory capacity (19).

Analysis showed the influence of age and gender as well, since the difference in weight loss mainly existed in adolescent boys. Developmental aspects could explain why the interactive influence of external eating and inhibition is especially important in an older age group. Adolescence is a period in which youngsters become more autonomous in deciding when and what to eat, and in which parental influence on the external food environment decreases (62, 63). In general, adolescents should become increasingly capable to manage this responsibility, because their self-control capacities are also meant to mature during this developmental phase (21). However, if their eating habits are food cue-related (high external eating) and impulsivity is high (high inhibition problems), they are less equipped to handle the challenge of resisting external cues in an obesogenic environment on their own (64). This does not imply that external eating and inhibition are unimportant in the earlier development of overweight, since previous studies indicate their relevance in children as well (10, 34). When considering the beta values in our study, inhibition problems in children were indeed also associated with weight loss, but not as strong as for adolescents. The fact that inhibition as a self-regulatory capacity is still developing in children, could account for the modest effects in this age group.

The effect of gender, which was mainly reflected in relationship to external eating, was not expected, since research does not consistently point to gender as an important predictor of external eating (4), inhibition (40), or weight loss (47). The role of gender proves to be more complex, and maybe additional factors have to be taken into account. For example, Burton and colleagues (4) could only discover gender differences in external eating when food craving and the specific types of food are taken into the equation. They claimed that men experienced external eating when confronted with all types of food, while women mainly showed external eating behavior towards specific types of (sweet) food. The gender effect could also be explained by attitudes towards food. The study of Havermans and colleagues (65), stated that women are more ambivalent towards unhealthy food, and mostly ignore bodily signals only in a state of negative emotion (4, 65). In children and adolescents, some studies did find more external eating patterns in boys, and more emotional eating in girls (10). To our knowledge however, this is the first study that is able to shed a light on gender differences in terms of the relationship between eating behavior to predict weight loss after MOT.

The results of this study have to be interpreted in line with its specific characteristics, strengths, and weaknesses. The sample size was large (n=572), revealing substantial insights for children and adolescents with extreme obesity. First, results showed that external eating and inhibition problems could play an important role in weight change in youngsters with extreme obesity, but only for subgroups. To test the hypothesis in different developmental subgroups, we ran the regression analyses separately for children (7–13) and adolescents (14–19). This subgroup approach led to a loss of power to detect significant predictors in all groups. A less conservative statistical approach, in which we ran the regression analysis^1^ on the total sample and included gender and age only in the interaction parameters, but not as main effects, showed more pronounced effects on both weight and weight loss. For example, subgroup differences then emerged in children as well. Future research could use these findings in new study paradigms that overcome power issues by choosing another type of analysis for allocating people to appropriate subgroups. For example, latent cluster analysis could reveal relevant profiles that can contribute to tailored interventions. Second, it has to be noted that the average adjusted BMI was extreme for all subgroups. This only allows to draw conclusions for a particular weight range, and does not necessarily generalize to girls, boys, children, and adolescents with overweight or modest obesity. Third, this study only focused on weight loss immediately after inpatient treatment. It is possible that the effects of external eating and inhibition changed at follow-up. After all, the youngsters returned to a less externally controlled and more obesogenic environment, in which inhibitory capacities are more extensively challenged. Future research should therefore include longer term assessment, to investigate whether changes in eating behavior and executive functions actually led to more weight control. Finally, this study only incorporated measures of self-reported external eating and parent-reported inhibition through questionnaires, and future research could benefit from a multi-method as well as a multi-informant approach of these concepts, perhaps more on an underlying trait level. Although a strength of this study is that T-scores were used to elevate the value of these indicators (being age and gender appropriate), lab-based eating paradigms (e.g. the Eating in the Absence of Hunger paradigm [31]), or experimental EF-tasks [e.g. Go/No Go (66)] could provide additional information on these concepts, even closer referring to actual behavior. However, they also not entirely capture the underlying traits of external eating and inhibition. Temperament parameters, such as effortful control, besides executive functions, the behavioral activation system (BAS) or reward responsivity may be a more accurate reflection of the psychological processes we were trying to grasp in this study (12). Also, inhibition was reported by parents. Although parents remain an important informant throughout development, youngsters are able to indicate their own problem behavior very well (67). The discrepancy between child and parent report on questionnaires especially appears in adolescents, in a way that adolescents generally indicate higher problems than their parents (67). Incorporating data from both parents and children, as well as through both questionnaires and objective behavior data, could enhance the methodological strength of future research on this topic (68).

In sum, we aimed at clarifying the relationship between maladaptive external eating behavior and weight, by scrutinizing differential effects of inhibition for children and adolescents as well as for boys and girls. We concluded that, under certain conditions, inhibition interfered in the relationship between external eating and weight status, but mainly in adolescent boys with good levels of inhibition predicting better outcome. Interventions aimed at increasing inhibition might be crucial to optimize treatment outcomes, and it will be important to take the existence of subgroups into account.

## Data Availability Statement

The datasets generated for this study are available on request to the corresponding author.

## Ethics Statement

The studies involving human participants were reviewed and approved by the Ethical Committee Faculty of Psychology and Educational Sciences. Written informed consent to participate in this study was provided by the participants’ legal guardian/next of kin. Written informed consent was obtained from the individual(s), and minor(s)’ legal guardian/next of kin, for the publication of any potentially identifiable images or data included in this article.

## Author Contributions

This study was conducted as a part a general research agreement between Ghent University and Zeepreventorium vzw, in which CB, AT, and AG were the principal collaborators. Furthermore, the WELCOME project, in which authors CB and LV were main applicants, enabled the first author (TN) to complete data analysis. All authors were involved in writing the manuscript and had final approval of the submitted versions.

## Funding

This study is funded by the Belgian FWO (Fonds voor Wetenschappelijk Onderzoek) as part of the TBM (Toegepast BioMedisch onderzoek) funding scheme (reference number T000316N).

## Conflict of Interest

The authors declare that the research was conducted in the absence of any commercial or financial relationship that could be construed as a potential conflict of interest.

## Footnotes

1 Results available on request to the corresponding author.
